# The Effect of Salts on the CO_2_ Reduction Product Distribution in an Aprotic Electrolyte

**DOI:** 10.1002/cphc.202400589

**Published:** 2024-11-08

**Authors:** Iris Burgers, Boris Wortmann, Amanda C. Garcia, Connor Deacon‐Price, Elena Pérez‐Gallent, Earl Goetheer, Ruud Kortlever

**Affiliations:** ^1^ Process and Energy Department Faculty of Mechanical Engineering Delft University of Technology Delft, Zuid-Holland 2628 CB The Netherlands; ^2^ Van ‘t Hoff Institute for Molecular Sciences University of Amsterdam Amsterdam, Noord-Holland 1098 XH The Netherlands; ^3^ Department of Sustainable Process and Energy Systems TNO Delft, Zuid-Holland 2628 CB The Netherlands

**Keywords:** Electrochemical CO_2_ reduction, Electrocatalysis, Organic solvents, Cation and anion effects, In-situ FTIR

## Abstract

Electrochemical CO_2_ reduction in non‐aqueous solvents is promising due to the increased CO_2_ solubility of organic‐based electrolytes compared to aqueous electrolytes. Here the effect of nine different salts in propylene carbonate (PC) on the CO_2_ reduction product distribution of polycrystalline Cu is investigated. Three different cations (tetraethylammonium (TEA), tetrabutylammonium (TBA), and tetrahexylammonium (THA)) and three different anions (chloride (Cl), tetrafluoroborate (BF_4_), and hexafluorophosphate (PF_6_)) were used. Chronoamperometry and in‐situ FTIR measurements show that the size of the cation has a crucial role in the selectivity. A more hydrophobic surface is obtained when employing a larger cation with a weaker hydration shell. This stabilizes the CO_2_^−^ radical and promotes the formation of ethylene. CO_2_ reduction in 0.7 M THACl/PC shows the highest hydrocarbon formation. Lastly, we hypothesize that the hydrocarbon formation pathway is not through C−C coupling, as the CO solubility in PC is very high, but through the dimerization of the COH intermediate.

## Graphical abstract

Electrochemical CO_2_ reduction in non‐aqueous solvents is promising due to their increased CO_2_ solubility. We find that the size of cations in the electrolyte has a crucial role on the product selectivity. A more hydrophobic surface, obtained when employing larger cations with a weaker solvation shell, stabilizes the CO_2_^−^ radical and promotes the formation of ethylene.

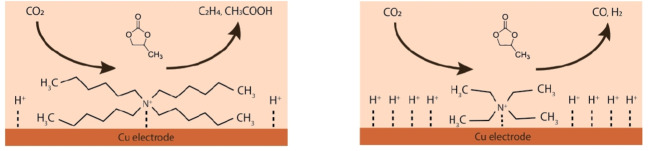

## Introduction

Electrochemical CO_2_ reduction is a promising sustainable pathway to convert flue gas into renewable fuels and chemicals by using renewable electricity[^1^, ^2^, ^3^] The CO_2_ reduction reaction (CO_2_RR) typically requires mild operating conditions and produces a variety of C_1_−C_3_ products.[^2^, ^4^] However, the reaction still suffers from large overpotentials, low current densities and a limited product selectivity.[^5^, ^6^, ^7^]

In CO_2_ reduction research, most commonly aqueous electrolytes are deployed.^[8]^ However, organic solvents are a promising alternative.[^8^, ^9^] The main advantages of using organic solvent are the relatively higher CO_2_ solubility compared to aqueous systems and the limited amounts of protons present, hence limiting the formation of H_2_ by the hydrogen evolution reaction (HER).[^7^, ^8^, ^9^] Frequently used organic solvents in CO_2_ reduction include acetonitrile (ACN), propylene carbonate (PC), and dimethylformamide (DMF).^[8]^ Several recent studies have explored CO_2_ reduction on Au electrodes using various organic solvents.[^10^, ^11^, ^12^, ^13^] For 2‐electron reduction products, such as CO, formate, and oxalate, promising results have been obtained on i. e. Pb, Ag and Pt electrodes.[^14^, ^15^, ^16^] Nonetheless, there is a relatively low amount of research conducted on the utilization of Cu electrodes, that are able to form hydrocarbons, in organic solvents.[^17^, ^18^, ^19^] All of these studies conclude that no hydrocarbons are formed while using a Cu electrode in aprotic solvent. Hydrocarbons are only observed when the aprotic electrolyte is altered with a proton donor such as phenol.^[20]^

Kumar et al.^[17]^ quantified the product distribution of CO_2_ reduction on a Cu electrode in dimethylformamide (DMF), n‐methyl‐2‐pyrrolidone (NMP), and acetonitrile (ACN), using 0.1 M tetrabutylammonium hexafluorophosphate (TBAPF_6_) as electrolyte. Different potential windows for the different solvents were used, investigating the difference in product distribution between −1.4 and −1.8 V vs. Ag/AgCl. For all three organic solvents, negligible amounts of hydrocarbons were detected. In DMF and NMP, the major product formed was oxalate, whereas hydrogen and formate were the main products found in ACN. The addition of water to the different organic solvents only increased the formation of hydrogen and formate with negligible hydrocarbon production. Hence, this study concludes that aqueous systems are the preferred solvent for the formation of hydrocarbons on a Cu electrode. No clear reasoning was given for the suppressed formation of hydrocarbons in organic solvent.

Deacon‐Price et al.^[18]^ recently studied the solvent effect in CO_2_ reduction on a nanostructured Cu electrode and compared this to a polycrystalline Cu electrode. A 0.1 M tetraethylammonium chloride (TEACl) in ACN was used as electrolyte, and CO_2_ reduction was performed at −2.0 V vs. Ag/Ag^+^. A small amount of hydrocarbons were observed, whereas the major product was CO. It is interesting to note that this differs from the observations of Kumar et al., who mainly observed formate production. As the only difference in these systems was the salt used, this indicates that the choice of cations and anions in organic solvents can have a significant effect on the product distribution. The addition of water to the organic solvent led to a slight increase in formate production and a minor increasing trend in hydrocarbon formation with increasing water concentration (up to 1000 mM). Kumar et al.^[17]^ investigated much larger water concentrations, hence they do observe an increase in hydrogen formation when increasing the water concentration (up to 5 % (v/v)). To better understand the competition between the hydrogen evolution reaction (HER) and CO_2_ reaction mechanism on the electrode surface, in‐situ FTIR measurements were performed. This revealed that the presence of CO_2_ completely suppressed the OH bending of water and lowered the OH stretching intensity, inhibiting the formation of hydrogen. However, the addition of water does not have any effect on the selectivity or activity of the CO_2_RR. Furthermore, it is hypothesized that CO desorption in acetonitrile is easier than in aqueous media. Hence the major product measured by the GC was found to be CO, as it detaches from the surface before a C−C bond can be formed, which is a crucial step in the pathway to hydrocarbons.

Lastly, in our previous work^[19]^ the product distribution of CO_2_ reduction using a Cu electrode in 0.7 M TEACl in PC as solvent was studied. As expected, negligible amounts of hydrocarbons were detected. The addition of water mainly increased the hydrogen formation. Increasing the operating temperature to 40 and 60 °C suppressed the hydrogen formation and increased the CO production. It was hypothesized that the hydrocarbon formation was suppressed due to the higher solubility of CO in PC compared to aqueous solvents leading to a low CO coverage on the catalyst surface and thus limiting C−C coupling.

We hypothesize that to further understand why previous studies for CO_2_ reduction in non‐aqueous electrolytes observe little to no hydrocarbons, the role of the electrolyte salt and its interaction with the catalytic surface needs to be better understood. The effect of the cation size on the selectivity of CO_2_ reduction on Cu electrodes in aqueous systems was already discussed in early studies from Murata and Hori.^[21]^ Smaller, strongly hydrated cations do not adsorb as easily on the electrode surface as larger cations. This changes the potential in the outer Helmholtz plane (OHP), hence effecting the selectivity of the CO_2_ reduction. It is well known that the larger cations, such as Cs^+^, lead to a higher selectivity towards hydrocarbons, compared to a smaller cations like Li^+^.[^6^, ^9^, ^22^, ^23^, ^24^] Furthermore, the anions are also known to have an effect on the selectivity, due to their buffering capacity and can serve as proton donors.^[8]^ Different studies have shown that the presence of halide anions, such as Cl^−^, Br^−^, and I^−^, has an effect on the Cu surface structure, and tend to steer the selectivity of the CO_2_ reduction on Cu electrodes towards C_2+_ products.[^6^, ^9^, ^25^]

The effect of cations and anions for CO_2_ reduction in organic solvents is less well understood. A study by Berto et al.^[12]^ compared the onset potential (obtained by cyclic voltammetry) for different NR_4_^+^ salts with varying alkyl chains. The onset potentials for all salts were very similar, hence they concluded that the cation did not have an catalytic role on the CO_2_ reduction and that the NR_4_^+^ does not have an electrostatic interaction with CO_2_ nor with the electrode surface area. This study did not further investigate the effect of the salts on the product distribution during CO_2_ reduction. Furthermore, Gomes et al.^[26]^ studied different tetrabutylammonium (TBA) salts in 1,2 dimethoxyethane (DME) and dimethyl sulfoxide (DMSO) for CO_2_ reduction on a Cu electrode. They showed that ion pair formation in DME is dependent on the anion type, whereas this is not the case for DMSO. Moreover, a decrease of ion pair formation increased the CO_2_ current density and CO Faradaic efficiency. Recent work by König et al.^[27]^ investigated the diffusion coefficients of CO_2_ in aprotic solvents and found that both the CO_2_ mass transport and the electrolyte solvent have an important effect on the product distribution towards CO or oxalate. Besides these studies, there is a limited amount of research available on the effect of the cation and anion on the product distribution for CO_2_ reduction on a Cu electrode.

In this study, a screening of the CO_2_ reduction performance of a copper electrode using several different organic salts dissolved in PC is reported. The product distribution was measured during 1 hour chronoamperometry (CA) experiments at −2.0 V vs. Ag/AgCl. Both the anion and the cation were varied, creating 9 different salt combinations. The length of the cation chain was increased from tetraethylammonium (TEA), to tetrabutylammonium (TBA), and tetrahexylammonium (THA). The anion was varied by using chloride (Cl), tetrafluoroborate (BF_4_), and hexafluorophosphate (PF_6_). We show that the size of the salt, both cation and anion, plays a major role in the observed product distribution. With in‐situ FTIR measurements the interaction of the salt, the PC and the catalytic surface were further investigated.

## Results and Discussion

### Electrolytic Performance

**Figure 1:**
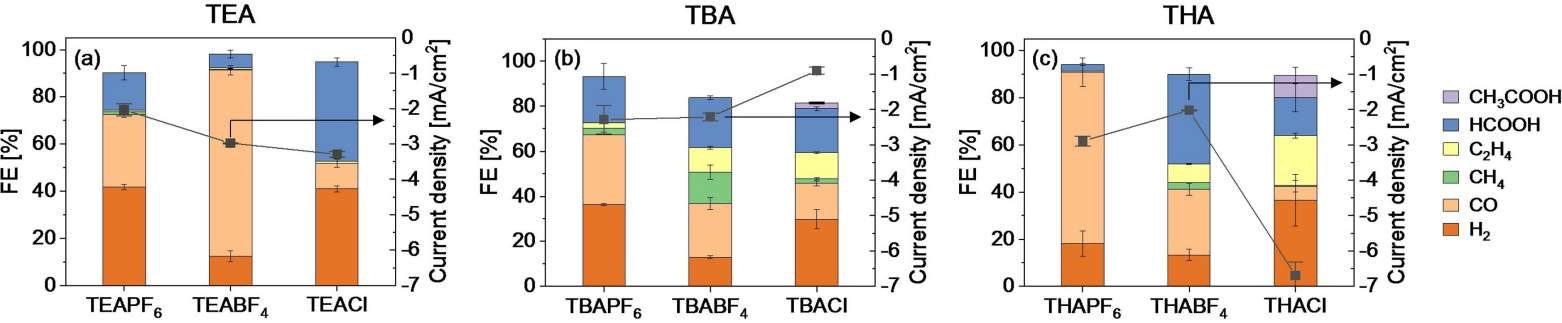
Faradaic efficiencies of CO_2_ reduction products during 1 hour chronoamperometry (CA) on a Cu electrode using PC electrolytes with different salts at −2.0 V vs. Ag/AgCl. All experiments were performed with 0.7 M salt concentrations. The results are grouped per anion used, with error bars indicating differences between duplicate measurements.

Figure 1 shows the faradaic efficiencies of products formed during 1 hour chronoamperometry (CA) experiments at −2.0 V vs. Ag/AgCl using a Cu electrode and the 9 different PC electrolytes. The investigated potential of −2.0 V vs. Ag/AgCl was chosen based on the optimum found in our earlier work on the CO_2_ reduction product distribution in a TEACl/PC electrolyte.^[19]^ All experiments were performed in a small H‐cell, which was deemed most appropriate to understand the mechanistic effects, with in‐line gas chromatography (GC) for the gas product analysis, and a constant salt concentration of 0.7 M. The results are grouped per anion, with the x‐axis as a function of the varying cations. Figure S1 in the supplementary information shows the results grouped per cation. Furthermore, Figure S4 shows the water concentration before and after electrolysis for each measured salt is presented. The salts naturally adsorb water from the atmosphere, hence it is difficult to start with a dry electrolyte without performing extra preparation steps. Therefore, the water concentration of the fresh electrolyte is measured to understand the starting conditions for the CA experiments. Due to the aqueous anolyte (0.5 M H_2_SO_4_), protons will diffuse through the Nafion membrane during the experiment. The water concentration is measured again after the electrolysis, to quantify the diffusion of water. By measuring the gaseous product distribution over time, we can understand the effect of the water in our system.

Figure 1 illustrates a notable disparity in the formation of hydrocarbon products across the nine tested salts. In the case of TEA as cation, the CO_2_ reduction products are consistently limited to CO and formate, irrespective of the anion employed. Hydrogen was also detected as result of the residual water and proton reduction reaction.^[28]^ Notably, the use of THACl yields the highest ethylene production (typical measured peak concentration of 165 ppm), while TBABF_4_ results in the highest methane yield (typical measured peak concentrations of 80 ppm). We hypothesize that the adsorption of the organic cations on the electrode surface increases the hydrophobicity of the surface, where the hydrophobicity of the surface increases with increasing alkyl chain lengths of the cation. Such a hydrophobic catalyst surface allows for limited available space for H^+^ to adsorb. Ethylene formation is favored in environments with low H^+^ availability, hence negligible amounts of ethylene were formed when using TEA salts, and the largest amount of ethylene was measured when using THA (22 % FE_C2H4_). The faradaic efficiency towards ethylene in THACl/PC is comparable to aqueous based electrolytes, where the observed faradaic efficiency towards ethylene is commonly around 30 %.[^6^, ^29^, ^30^] Methane formation is favored with a higher H^+^ coverage.^[6]^ Therefore, the faradaic efficiency of methane was higher for the TBA salts than for the THA salts. However, since for the TBA and THA cations there was only ethylene production when combined with BF4^−^ or Cl^−^ anions, the production of hydrocarbons cannot solely be dependent on the size of the cation.

When looking at the anion effect, the systems with salts using PF_6_^−^ as anion showed negligible or no hydrocarbons production. BF_4_^−^ anions only showed decent amounts of methane and ethylene when combined with a larger cation, such as TBA and THA. Cl^−^ anions showed a similar trend, where hydrocarbons were observed when TBACl or THACl was used in the PC solvent during CO_2_ reduction. When using TBACl and THACl, some acetic acid was measured, which was not detected when using any of the other salts. However, with THACl no methane was measured suggesting that with THACl the C_1_ pathway forming methane is suppressed in favor of the C_2_ pathway. Furthermore, the salts with a Cl^−^ anion show the highest water concentration in the electrolyte, whereas the salts with PF_6_^−^ anion have the lowest water concentration in the electrolyte (see Figure S4). Cl^−^ based salts are better soluble in water than the organic based BF_4_ and PF_6_ salts, which can explain the larger amount of water present in the solvents using Cl^−^ salts. The high concentrations of water when Cl^−^ anions were used can explain why more formate was formed, in comparison to the PF_6_^−^ and BF_4_^−^ anions. Despite this, an increased formate faradaic efficiency was only observed with TEACl and TBACl and not with the THACl.

Additionally, chronoamperometry experiments with a non‐aqueous catholyte and anolyte were conducted to confirm that the observed change in product distribution is mainly due to the salt used and not due to the unregulated water concentration caused by diffusion through the membrane. However, due to the Nafion membrane used which is activated and stored in water, the water in the system cannot be completely eliminated. TEACl and THACl salts were tested, using a PC electrolyte as anolyte and catholyte. Figure S5 shows the faradaic efficiencies of the CO_2_ reduction products. Comparable results were obtained, confirming that the nature of the salt present in the electrolyte is indeed controlling the selectivity of the CO_2_RR and controlling whether ethylene is produced. Moreover, as a decrease in ethylene faradaic efficiency is observed when using a PC based anolyte, caused by the lower concentration of water (see Figure S6) as proton donor which is required in non‐aqueous electrolytes to obtain significant amounts of ethylene.

### In‐situ FTIR Spectroscopy

**Figure 2:**
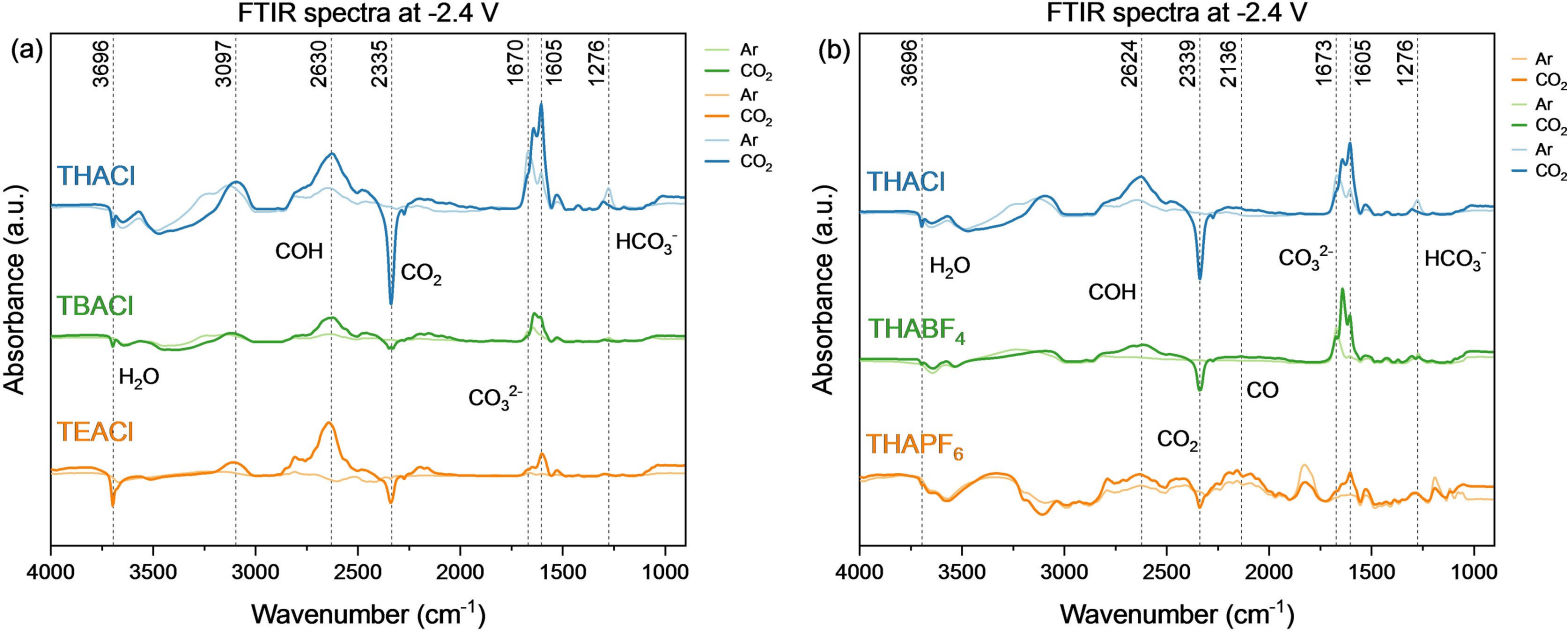
FTIR spectra at −2.4 V vs. Ag/AgCl for the different salts (0.7 M) dissolved in PC, for Ar and CO_2_ saturated conditions, using a Cu electrode. (a) Spectra for electrolytes using THACl, TBACl, and TEACl in PC. (b) Spectra for electrolytes using THACl, THABF_4_, and THAPF_6_ in PC.

To better comprehend how the cations and anions interact with the catalytic surface during CO_2_ reduction, in‐situ FTIR measurements were performed. Figure 2 shows the spectra at −2.4 V vs. Ag/AgCl for both Ar and CO_2_ saturated conditions. The full spectra can be found in the Supporting Information (Figure S7 and Figure S8). This potential was chosen as it clearly shows the different bands for all measured conditions. The effect of the anion on the CO_2_ reduction product distribution is tested by investigating THAPF_6_/PC, THABF_4_/PC, and THACl/PC, and the effect of the cation by comparing TEACl/PC, TBACl/PC, and THACl/PC.

In Figure 2(a) a negative band is observed at 2335 cm^−1^, which corresponds to CO_2_ being consumed during the CO_2_ reduction reaction.[^18^, ^28^] A relatively larger negative band is indicative for a higher CO_2_ consumption, which corresponds well to the total partial current density for CO_2_ reduction for the different salts (see SI Figure S3 and Figure S8).

Furthermore, at 1276 cm^−1^ a growing positive band is observed (see Figure 2(a)) which is attributed to bicarbonate formation.^[31]^ The bands in between 1673–1605 cm^−1^ are likely related to the carbonate concentration at the surface.^[28]^ It is hypothesized that the increase in (bi)carbonate at the surface is related to an increased hydrocarbon production, because an increase in the amount of (bi)carbonates indicates a higher local alkalinity at the surface.^[8]^ Comparing to the product distribution (as given in Figure 1), the largest amount of hydrocarbons were produced when using THACl, which directly corresponds to the highest intensity of the bands around the carbonate and bicarbonate wavelength. The least amount of hydrocarbons were formed when using THAPF_6_. In the spectrum of CO_2_ saturated 0.7 M THAPF_6_/PC (dark orange line in Figure 2(b)), there is very little to no band present at these wavenumbers.

Furthermore, the Ar saturated spectra (see Figure S7 in the SI), also show (bi)carbonate bands. It is assumed that in the FTIR blank control experiments (using an Ar saturated electrolyte), these bands correspond to the degradation of propylene carbonate, forming (bi)carbonate at these negative potentials. Due to the high OH^−^ concentration on the surface, the alkaline pH catalyzes the chemical degradation process of propylene carbonate into (bi)carbonate.^[32]^ Hence, in the FTIR spectrum, the breakdown of PC is already visible at a lower potential compared to the onset potential measured in the blank CV experiments (see SI Figure S9 and Figure S10).

There are multiple pathways through which hydrocarbons can be formed. The first is through the C_1_ pathway, where *COH is a key intermediate.[^4^, ^6^] Dimerization of intermediates in this pathway leads to the production of both methane and C_2_ products. Second, a C−C bond can be formed through the C_2_ pathway, where two adsorbed *CO(H) molecules dimerize. There has been discussion regarding the exact dimerization reaction, as both *CO and *COH have been identified as reactants for this dimerization.[^33^, ^34^] Typically, a band corresponding to adsorbed *CO is expected around 2136 cm^−1^, however such a band is not observed in any of the spectra. This can be due to the limited sensitivity of FTIR for measuring adsorbed *CO on a Cu electrode surface. A similar observation was made by Deacon‐Price et al.,^[18]^ where they studied CO_2_ reduction in acetonitrile. Additionally, they observed a higher FE% of CO, which was detected by gas chromatograph, in comparison to an aqueous solvent, suggesting indeed that CO do not adsorb strong on Cu surface in less protic solvent.

In contrast, a clear band is observed around 2630 cm^−1^, that is attributed to a *COH intermediate. The presence of this intermediate on the surface suggests that the reaction pathway to form hydrocarbons is either via the C_1_ pathway, where dimerization of intermediates occurs to form ethylene, or through the dimerization of *COH intermediates, possibly with co‐adsorbed CO.

Lastly, the negative bonds at 3696 cm^−1^ are related to OH^−^ stretching of the water present in the electrolytes. The salts with Cl^−^ anions contained the most amount of water, which correlates with the intensity of the negative band related to OH^−^ stretching. There is no negative band observed near 1628 cm^−1^, which typically corresponds to OH^−^ bending of water.^[18]^ However, the total amount of water present at the end of the FTIR measurement will be comparable to the water concentration measured before electrolysis, since no water crossover from the anolyte can happen during the experiment. Furthermore, this band is overlapping with the (bi)carbonate bands, which could also be the reason why this is not clearly visible.

## Conclusions

This study shows that the electrolyte, and more concretely the type of salt used, plays a crucial role in determining the product distribution during CO_2_ reduction in propylene carbonate. We conclude that the size of the cation strongly effects the product formation. A larger cation, with an increasing alkyl chain length, creates an increasingly hydrophobic surface which promotes the formation of ethylene. Hence, using THACl/PC results in the highest ethylene formation. Furthermore, based on the FTIR results, we conclude that a higher (bi)carbonate concentration near the electrode surface is related to a higher hydrocarbon production. The differences in (bi)carbonate concentration at the surface is related to the local pH at the electrode. This affects the observed product distribution, as a higher local pH promotes C_2+_ hydrocarbon production. The observed concentration of (bi)carbonate at the surface is highly dependent on the dissolved salt in the electrolyte. The THACl salt shows the highest amount of (bi)carbonate at the surface, which indeed corresponds to the highest amount of hydrocarbons formed during the CA experiments. Lastly, we hypothesize that the reaction pathway for hydrocarbon formation in a PC electrolyte occurs dominantly through the *COH intermediate, either through the C_1_ or C_2_ pathways, irrespective of the salt used. This is supported by FTIR spectra where a large band corresponding to COH was measured. Hence, the salt has a major role in the product selectivity of CO_2_ reduction in PC. The highest amount of ethylene (22 % FE_C2H4_) was achieved using 0.7 M THACl in PC.

## Experimental Section

### Electrochemical Cell

All chronoamperometry experiments were performed using a two‐compartment H‐cell, based on the design by Lobaccaro et al.^[35]^ The cell was stored in 20 % v/v nitric acid to prevent any contamination and rinsed thoroughly with MilliQ water before use. A copper foil (Sigma Aldrich 99.999 %, 1 mm thickness, 2.5 cm×2.5 cm) was used as working electrode. Initial sanding was performed (up to grain size 2000), followed by mechanical polishing (up to 1 μm grade diamond paste). Before each experiment the copper foil was electropolished using phosphoric acid solution (85 % in H_2_O, Sigma Aldrich) at 2.1 V versus a graphite rod for 3 min, based on a procedure by Kuhl et al.^[30]^ A platinum counter electrode (MaTeck 99.99 %, 0.1 mm thickness, 2.5 cm×2.5 cm) and a leak‐free Ag/AgCl reference electrode (Innovative Instruments, LF‐1‐45) were used. A cation exchange membrane (Nafion‐117) was used to separate the anolyte and catholyte compartment. The anolyte used was a 0.5 M aqueous H_2_SO_4_ (Sigma Aldrich) and catholyte were prepared using propylene carbonate (Sigma Aldrich, anhydrous 99.7 %) with either 0.7 M TEACl (98 %, Sigma Aldrich), TBACl (97 %, Sigma Aldrich), THACl (96 %, Sigma Aldrich), TEABF_4_ (99 %, Sigma Aldrich), TBABF_4_ (99 %, Sigma Aldrich), THABF_4_ (97 %, Sigma Aldrich), TEAPF_6_ (99 %, Sigma Aldrich), TBAPF_6_ (98 %, Sigma Aldrich) or THAPF_6_ (98 %, Alfa chemistry). A potentiostat (SP‐200, BioLogic) was used perform the cyclic voltammetry and chronoamperometry experiments. A continuous CO_2_ gas stream of 8 sccm was supplied at the bottom of the cathode compartment. The outlet was connected to an inline gas chromatograph (Compact GC 4.0, Interscience), equipped with two thermal conductivity detectors (TCD) and one flame ionization detector (FID), that measured the gaseous products formed in the cathode compartment with 2 minute intervals. The reported faradaic efficiencies are calculated as the average value of the stable period during the last 15 minutes of the experiment. Liquid samples were taken at the end of the 1 hour experiment for which the product analysis was done by high pressure liquid chromatograph (HPLC,1290 Infinity II, Agilent). For HPLC analysis, 5 μl of the catholyte solution was injected on two Aminex HPX‐87H columns (Biorad) placed in series. The columns were heated to 60 °C, using an eluent containing 1 mM H_2_SO_4_ in ultrapure water and a refractive index detector (RID) for the detection of products. Coulometric Karl Fischer titration (Metrohm 756 KF Coulometer) was used for the water content measurement before and after the experiment.

### Fourier Transform Infrared Spectroscopy

The FTIR experiments were performed using a Bruker Vertex 80 V IR spectrometer in combination with an Autolab PGSTAT12 potentiostat. A three electrode spectro‐electrochemical cell configuration was used in combination with a 60° CaF_2_ prism pressed to the bottom of the cell.[^28^, ^36^] A polycrystalline Cu foil working electrode (MaTeck 99.99 %), platinum coil counter electrode (MaTeck 99.99 %), and Ag/Ag+ reference electrode (Alvatek) were used. Spectra were collected in a thin‐film configuration in the wavenumber range 4000–900 cm^−1^ by varying the potential from −1.0 V to −2.5 V vs. Ag/AgCl with steps of 0.1 V under Argon and CO_2_ saturated conditions (see SI Figure S7 and Figure S8). A reference spectrum was obtained and subtracted from the spectra at the measured potentials. The spectra are presented as absorbance spectra, according to A=‐$\log\frac{R}{R_{0}}$ where R and R_0_ is the reflectance corresponding to the single beam spectra obtained at the sample and reference potential, respectively. Therefore, negative bands correspond to consumed species on the surface, whereas positive bands correspond to the formation of species. Prior to the experiment, the Cu electrode was electropolished using the same method as described in the previous section, except the polishing was done for 15 seconds only.

## Conflict of Interests

The authors declare no conflict of interest.

1

## Supporting information

As a service to our authors and readers, this journal provides supporting information supplied by the authors. Such materials are peer reviewed and may be re‐organized for online delivery, but are not copy‐edited or typeset. Technical support issues arising from supporting information (other than missing files) should be addressed to the authors.

Supporting Information

## Data Availability

The data that support the findings of this study are available from the corresponding author upon reasonable request.
