# Supplementary material for: The Effect of Salts on the CO2 Reduction Product Distribution in an Aprotic Electrolyte
Source: Chemphyschem. 2024 Nov 8;25(24):e202400589. doi: 10.1002/cphc.202400589 (PMC11648821; doi:10.1002/cphc.202400589)
Supplement: Supplementary file 1 — Supporting Information [file CPHC-25-e202400589-s001.pdf]

ChemPhysChem

Supporting Information

The Effect of Salts on the CO_2 Reduction Product Distribution in an Aprotic Electrolyte

Iris Burgers, Boris Wortmann, Amanda C. Garcia, Connor Deacon-Price, Elena Pérez-Gallent, Earl Goetheer, and Ruud Kortlever*

Supporting Information to:

The Effect of Salts on the CO_2 Reduction Product Distribution in an Aprotic Electrolyte

Iris Burgers,[a] Boris Wortmann,[a] Amanda C. Garcia,[b] Connor Deacon-Price,[b] Elena Pérez-Gallent,[c] Earl Goetheer,[a] Ruud Kortlever*[a]

[a] I.A.E. Burgers, B. Wortmann, E.L.V. Goetheer, R.Kortlever
Process and Energy Department
Delft University of Technology
Delft, Zuid-Holland, 2628 CB, The Netherlands
E-mail: r.kortlever@tudelft.nl

[b] A.C. Garcia, C. Deacon-Price
Van 't Hoff Institute for Molecular Sciences
University of Amsterdam
Amsterdam, Noord-Holland, 1098 XH, The Netherlands

[c] E. Pérez-Gallent
Department of Sustainable Process and Energy Systems
TNO
Delft, Zuid-Holland, 2628, The Netherlands

Table of Figures

Figure S1 CO_2 reduction product distribution after 1 hour chronoamperometry (CA) on a Cu electrode using PC solvent with different salts at 0.7 M grouped per cation, at -2.0 V vs. Ag/AgCl. Error bars indicate differences between duplicate measurements **2**

Figure S2 CO_2 reduction product distribution after 1 hour chronoamperometry (CA) on a Cu electrode using PC solvent with different salts at different concentrations at -2.0 V vs. Ag/AgCl. Error bars indicate differences between duplicate measurements. **2**

Figure S3 Partial current densities after 1 hour chronoamperometry (CA) on a Cu electrode using PC solvent with different salts at 0.7 M at -2.0V vs. Ag/AgCl. Error bars indicate differences between duplicate measurements **2**

Figure S4 Water concentrations (ppm) of the different electrolytes used during CO_2 reduction at -2.0 V vs. Ag/AgCl before and after electrolysis. 2

Figure S5 Faradaic efficiencies of CO_2 reduction products during 1 hour chronoamperometry on a Cu electrode using 0.7 M TEACl in PC and 0.7 M THACl in PC as catholyte combined with either an aqueous 0.5 M H_2SO_4 anolyte or similar PC anolyte. **2**

Figure S6 Water concentration (ppm) of the different electrolytes used during CO_2 reduction at -2.0 V vs. Ag/AgCl before and after electrolysis, comparing an aqueous H_2SO_4 and non-aqueous propylene carbonate anolyte. **2**

Figure S7 FTIR spectra from -1.1 V to -2.6 V vs. Ag/AgCl for the different salts (0.7 M) dissolved in PC, for Ar saturated conditions, using a Cu electrode. **2**

Figure S8 FTIR spectra from -1.1 V to -2.6 V vs. Ag/AgCl for the different salts (0.7 M) dissolved in PC, for CO_2 saturated conditions, using a Cu electrode **2**

Figure S9 Cyclic voltammetry for the different TEA and TBA salts at varying concentrations in PC, both for Argon and CO_2 saturated electrolytes. **2**

Figure S10 Cyclic voltammetry for the different THA salts and concentration in PC, for Argon and CO_2 saturated electrolytes. **2**

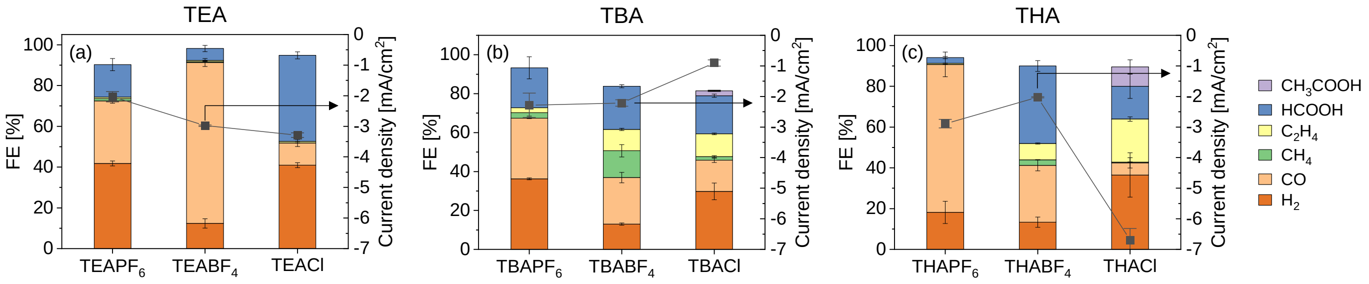

Figure S1 CO_2 reduction product distribution after 1 hour chronoamperometry (CA) on a Cu electrode using PC solvent with different salts at 0.7 M grouped per cation, at -2.0 V vs. Ag/AgCl. Error bars indicate differences between duplicate measurements

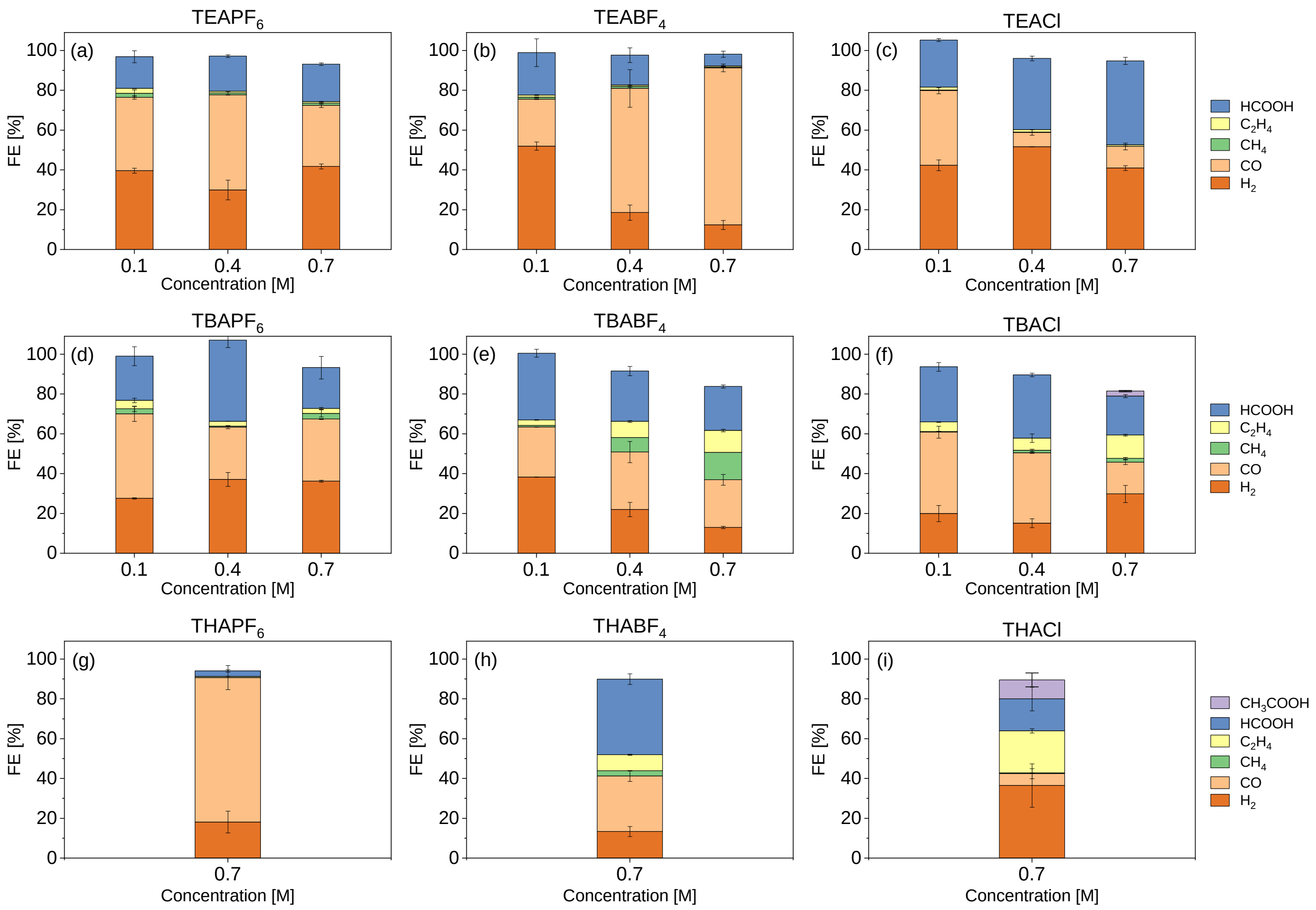

Figure S2 CO_2 reduction product distribution after 1 hour chronoamperometry (CA) on a Cu electrode using PC solvent with different salts at different concentrations at -2.0 V vs. Ag/AgCl. Error bars indicate differences between duplicate measurements.

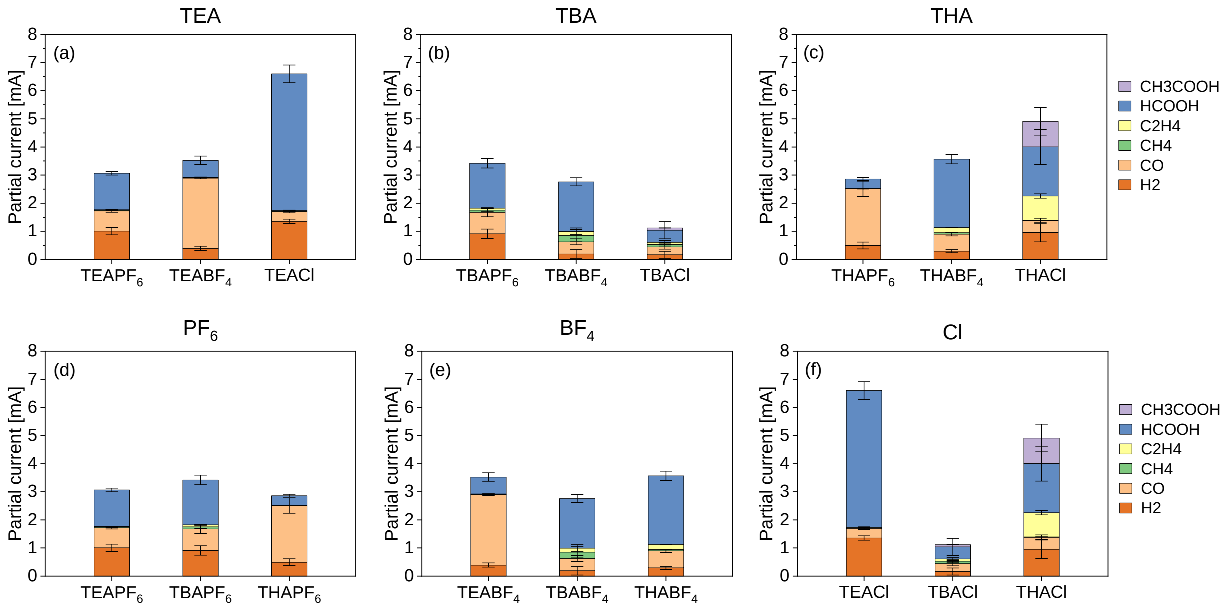

Figure S3 Partial current densities after 1 hour chronoamperometry (CA) on a Cu electrode using PC solvent with different salts at 0.7 M at -2.0V vs. Ag/AgCl. Error bars indicate differences between duplicate measurements

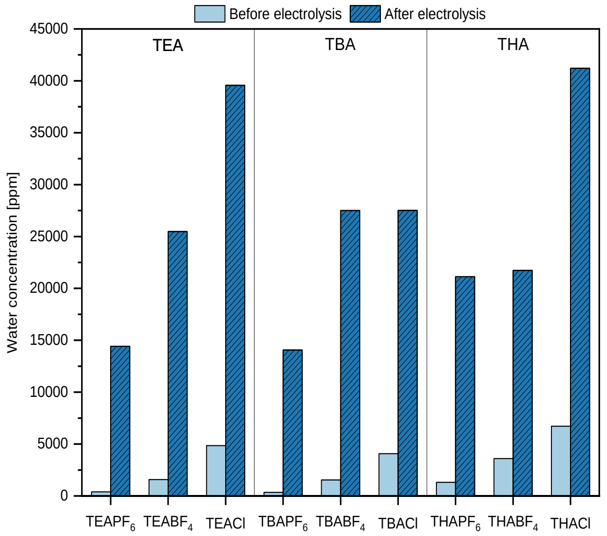

Figure S4 Water concentrations (ppm) of the different electrolytes used during CO_2 reduction at -2.0 V vs. Ag/AgCl before and after electrolysis.

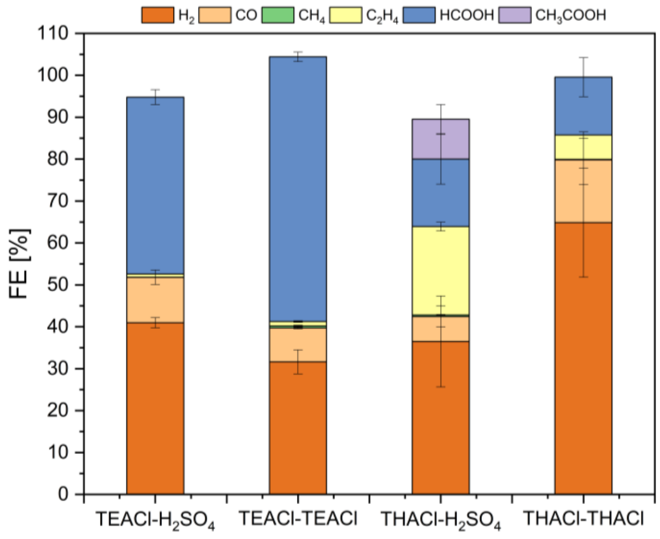

Figure S5 Faradaic efficiencies of CO_2 reduction products during 1 hour chronoamperometry on a Cu electrode using 0.7 M TEACl in PC and 0.7 M THACl in PC as catholyte combined with either an aqueous 0.5 M H_2SO_4 anolyte or similar PC anolyte. The error bars indicate differences between duplicate measurements.

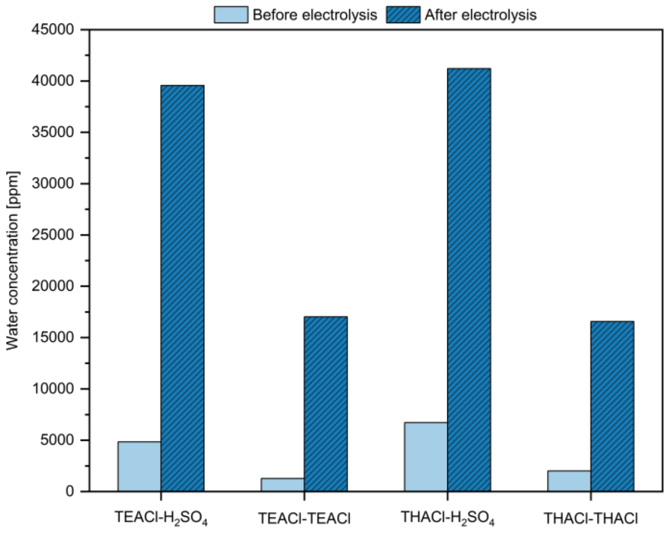

Figure S6 Water concentration (ppm) of the different electrolytes used during CO_2 reduction at -2.0 V vs. Ag/AgCl before and after electrolysis, comparing an aqueous H_2SO_4 and non-aqueous propylene carbonate anolyte.

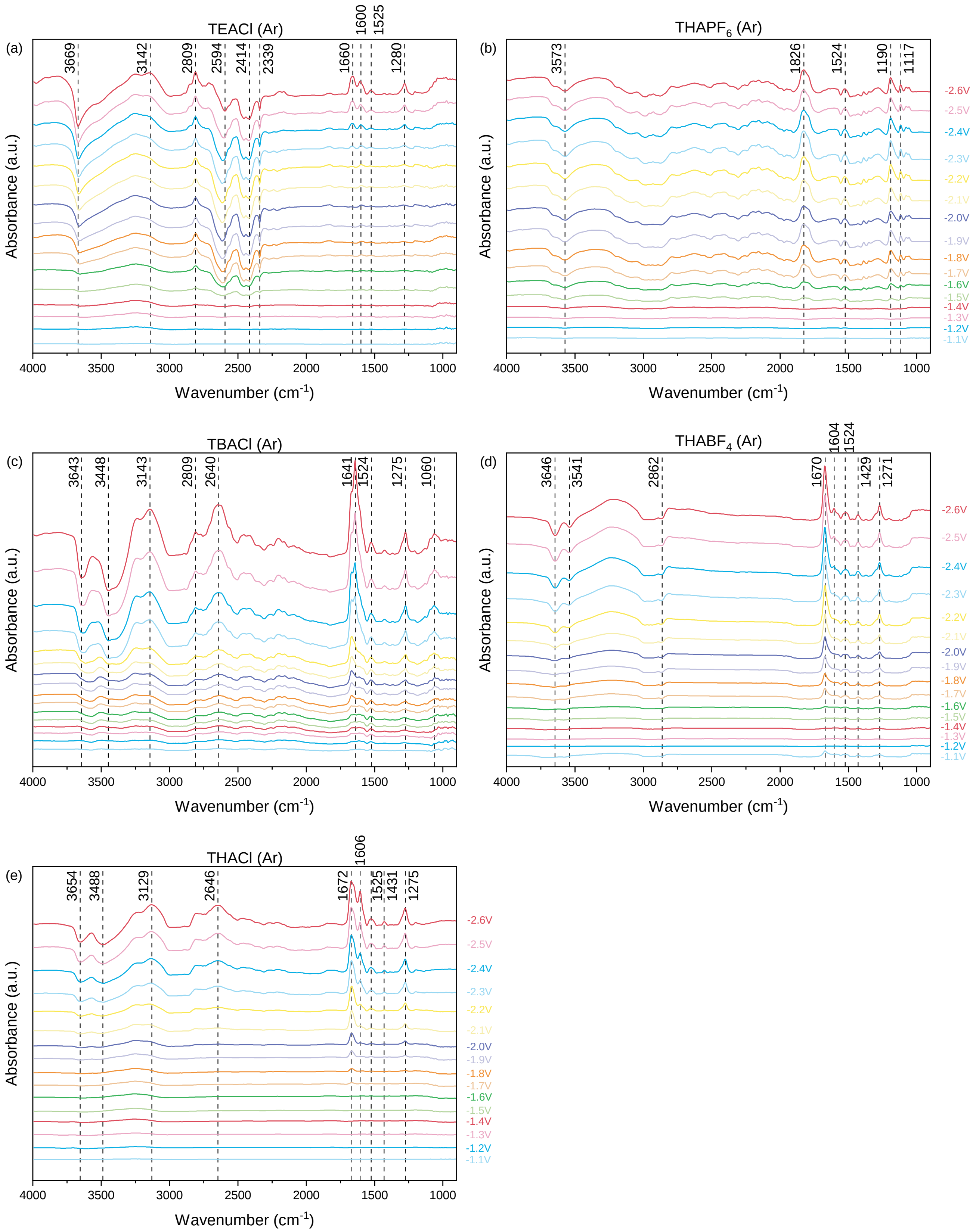

Figure S7 FTIR spectra from -1.1 V to -2.6 V vs. Ag/AgCl for the different salts (0.7 M) dissolved in PC, for Ar saturated conditions, using a Cu electrode.

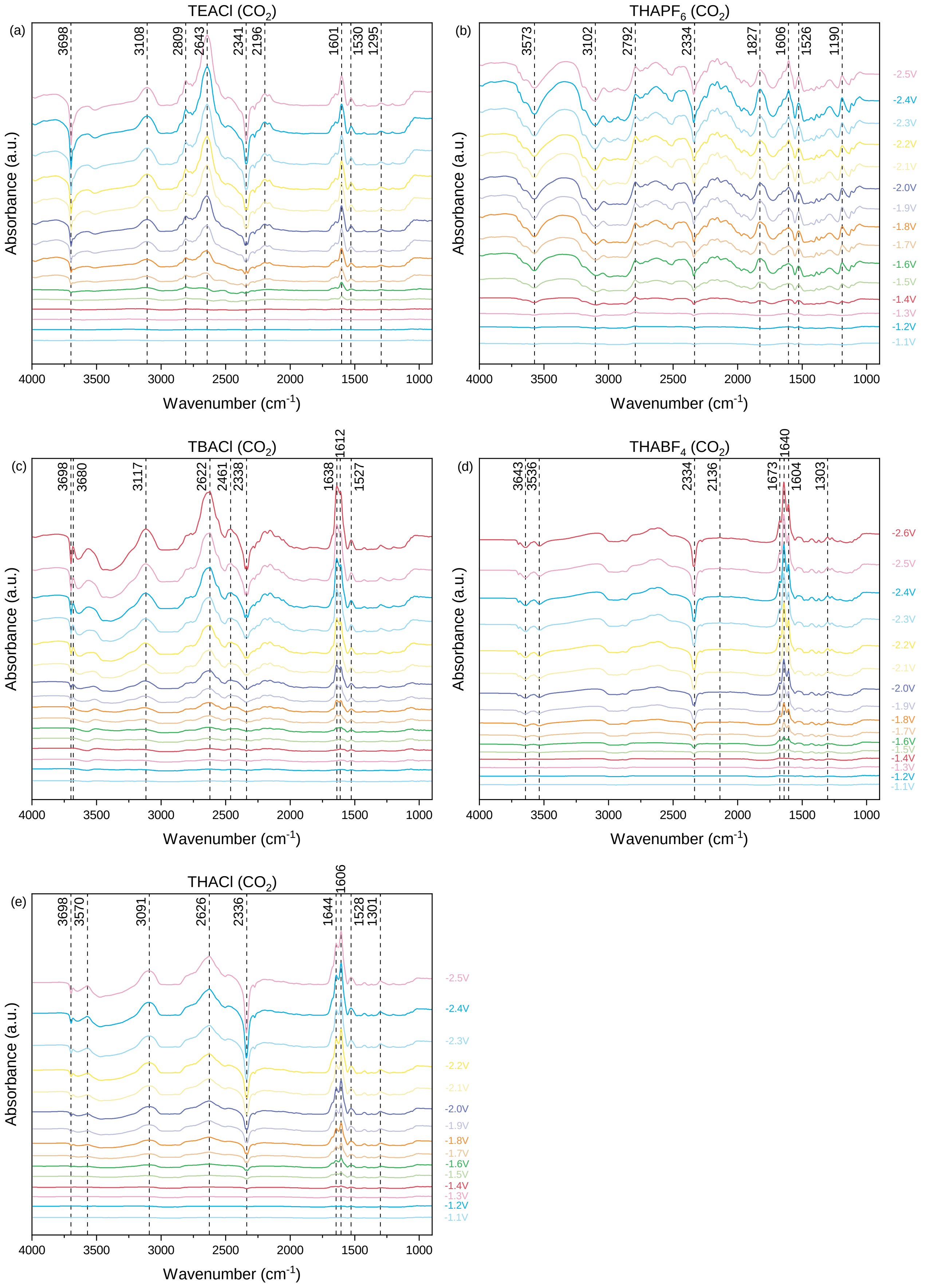

Figure S8 FTIR spectra from -1.1 V to -2.6 V vs. Ag/AgCl for the different salts (0.7 M) dissolved in PC, for CO_2 saturated conditions, using a Cu electrode

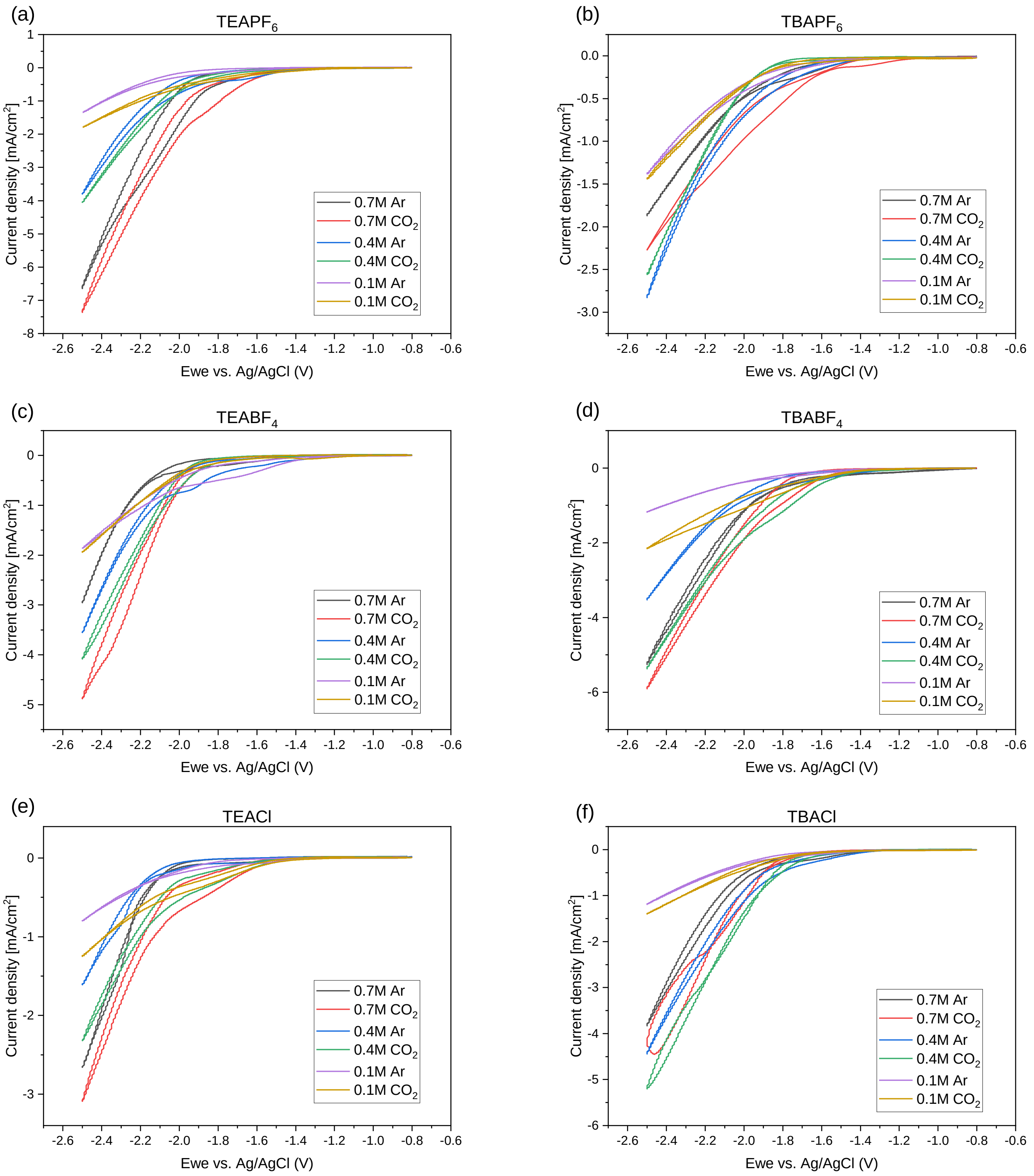

Figure S9 Cyclic voltammetry for the different TEA and TBA salts at varying concentrations in PC, both for Argon and CO_2 saturated electrolytes.

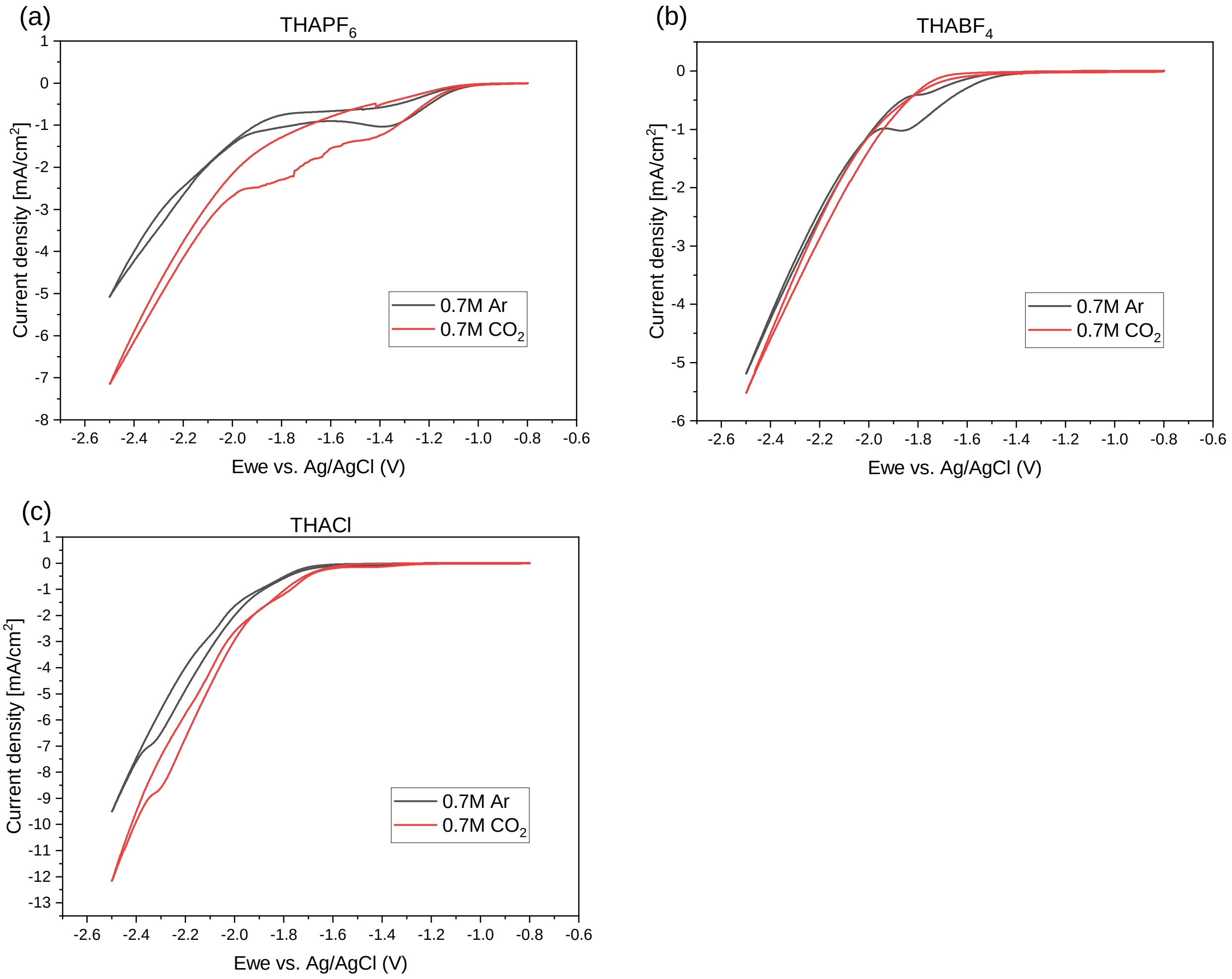

Figure S10 Cyclic voltammetry for the different THA salts and concentration in PC, for Argon and CO_2 saturated electrolytes.
